# Relationship between Degree of Dependency and Hospitalization Time of Surgical Patients*

**DOI:** 10.17533/udea.iee.v41n1e10

**Published:** 2023-03-14

**Authors:** José Ander Asenjo-Alarcón, Luz Elita Vergara Cieza

**Affiliations:** 1 Degree in Nursing, Epidemiologist, PhD. Professor. Faculty of Health Sciences, Universidad Nacional Autónoma de Chota, Chota, Cajamarca, Peru. E-mail: ander1213@hotmail.com. Universidad Nacional Autónoma de Chota Faculty of Health Sciences Universidad Nacional Autónoma de Chota Chota Cajamarca Peru ander1213@hotmail.com; 2 Bachelor of Nursing. Faculty of Health Sciences, Universidad Nacional Autónoma de Chota, Chota, Cajamarca, Peru. E-mail: elitavergara10@gmail.com Universidad Nacional Autónoma de Chota Faculty of Health Sciences Universidad Nacional Autónoma de Chota Chota Cajamarca Peru elitavergara10@gmail.com

**Keywords:** surgical intervention, postoperative care, mobility limitation, hospitalization, hospitals, Peru, intervención quirúrgica, cuidados posoperatorios, limitación de la movilidad, hospitalización, hospitales, Perú, Intervenção cirúrgica, cuidados pós-operatórios, limitação da mobilidade, hospitalização, hospitais, Peru, Dependiente del cuidado de enfermería

## Abstract

**Objective.:**

This work sought to establish the relationship between the degree of dependency with hospitalization time of patients intervened surgically in a regional hospital of Peru.

**Methods.:**

The study was analytical, cross-sectional with retrospective data collection, studying 380 patients treated in the surgical service at Regional hospital Docente in the municipality of Cajamarca (Peru). The patients’ demographic and clinical information was obtained from the daily care records in the hospital’s surgery service. The univariate description was conducted through absolute and relative frequencies and confidence intervals for proportions at 95%; for the association between the degree of dependency and hospitalization time Log Rank (Mantel-Cox) - Chi-square was applied, as well as the Kaplan-Meier survival analysis, with statistical significance of *p*<0.05.

**Results.:**

The study had 53.4% male patients, with mean age of 35.3 years, referrals from operating room (64.7%), surgery specialty (66.6%) and the most-frequent surgical intervention was appendectomy (49.7%). Mean hospitalization time was 10 days; 88.1% of the patients had grade-II dependency. The degree of patient dependency had a significant impact on the days of post-surgery hospitalization with direct relationship between both variables (*p*=0.038).

**Conclusion.:**

Hospitalization time is determined by the degree of dependency of patients subjected to a surgical intervention; thereby, it is fundamental to anticipate all the necessary resources for proper care management.

## Resumen

**Objetivo.:**

Establecer la relación entre el grado de dependencia con el tiempo de hospitalización de pacientes intervenidos quirúrgicamente en un hospital regional de Perú.

**Métodos.:**

El estudio fue analítico, transversal con toma retrospectiva de la información. Se estudiaron 380 pacientes atendidos en el servicio de cirugía del Hospital Regional Docente de Cajamarca (Perú). La información demográfica y clínica de los pacientes se obtuvo del registro de atenciones diarias del servicio de cirugía del hospital. La descripción univariada se hizo a través de frecuencias absolutas y relativas e intervalos de confianza para proporciones al 95% y para la asociación entre el grado de dependencia y el tiempo de hospitalización se aplicó Log Rank (Mantel-Cox) - Chi-cuadrado y el análisis de supervivencia de Kaplan-Meier, con una significancia estadística de *p*<0.05.

**Resultados.:**

El 53.4% de pacientes fueron varones, con edad promedio de 35.3 años, referidos de sala de operaciones (64.7%), especialidad de cirugía (66.6%) y la intervención quirúrgica más frecuente fue la apendicectomía (49.7%). El tiempo medio de hospitalización fue de 10 días, el 88.1% de pacientes tuvieron dependencia de grado II. El grado de dependencia de los pacientes repercutió significativamente en los días de hospitalización pos-cirugía con una relación directa entre ambas variables (*p*=0.038).

**Conclusión.:**

El tiempo de hospitalización está determinado por el grado de dependencia que presentan los pacientes que son sometidos a una intervención quirúrgica, por ello es fundamental la previsión de todos los recursos necesarios para la gestión adecuada del cuidado.

## Resumo

**Objetivo.:**

Estabelecer a relação entre o grau de dependência e o tempo de internação de pacientes operados em um hospital regional do Peru.

**Métodos.:**

O estudo foi transversal analítico com obtenção retrospectiva de informações. Foram estudados 380 pacientes atendidos no serviço de cirurgia do Hospital Universitário Regional do município de Cajamarca (Peru). As informações demográficas e clínicas dos pacientes foram obtidas da ficha de atendimento diário do serviço de cirurgia do hospital.

**Resultados.:**

53.4% dos doentes eram do sexo masculino, com idade média de 35.3 anos, provenientes do bloco operatório (64.7%), especialidade cirúrgica (66.6%) e a intervenção cirúrgica mais frequente foi a apendicectomia (49.7%). O tempo médio de internação foi de 10 dias, 88.1% dos pacientes apresentavam dependência grau II. De acordo com o teste de Log Rank (Mantel-Cox) o grau de dependência dos pacientes teve impacto significativo nos dias de internação após a cirurgia com relação direta entre ambas as variáveis (*p*=0.038).

**Conclusão.:**

O tempo de internação é determinado pelo grau de dependência apresentado pelos pacientes que se submetem à cirurgia, portanto, é essencial fornecer todos os recursos necessários para o gerenciamento adequado de seus cuidados.

## Introduction

Surgical interventions are frequently practiced to reverse a pathology, improve an individual’s life conditions, or prevent the progression of a disease; individuals of any age can be subjected to them, after evaluating their physical and physiological state. All surgical interventions imply some degree of risk, which will be evaluated in relation to the underlying disease, the surgical strategy used, and the hospital process, besides being aspects that also determine the recovery time during the postoperative period.^1^ Likewise, the surgical process brings along dependency on nursing care in patients, which must be aimed at satisfying care needs with human sense, quality, excellence, and achieving their wellbeing.^2^ Said conditions become difficult to fulfill with patients with greater needs, or who have a prolonged hospital stay or their degree of dependency is higher;^3^ moreover, when the nursing resource is insufficient to cover the demand of patients or the number of beds assigned to care exceeds the capacities of the professional, the service no longer meets quality characteristics.^4^ In this sense, estimating the hospitalization days of patients - bearing in mind the factors that can interfere in the process, will permit improving the hospital management system. 

The hospitalization period may be influenced by factors inherent or extrinsic to patients undergoing surgical intervention, among them age, given that elderly adults have longer hospital stays, and diseases, like arterial hypertension, cancer, prolong it.^5^ These factors, added to the outcome of the surgical process, condition the degree of dependency patients will have after the surgery and the needs for nursing care. Likewise, it is also important to estimate during the presurgical phase the degree of patient dependency; given that if they are functionally independent, they have greater probabilities of being so after the surgery, however, if they have difficulties in carrying out daily life activities or to assist themselves independently, such can be deteriorated during the rehabilitation. For this reason, the risk-benefit assessment of the surgery and the health staff’s foreseeing incidences, gains relevance upon its planning to achieve the independence of patients in the shortest time and under the best conditions.^6^

Patients with comorbidities or sensitive conditions known prior to the surgery have prolonged hospitalization time and greater demand for nursing care, according to their degree of dependency. On average, one third of patients undergoing elective abdominal surgery fail to restore normal mobility after four weeks of the surgical intervention, due in large part to extensive surgeries, open surgeries, post-surgery complications, and reduced mobility levels during the postoperative.^7^ These situations can be handled adequately and in timely manner if the necessary resources are provided to favor incorporating patients to their daily activities. 

The contextualized education and complete information provided by the nursing professional to patients on the guidelines to follow before, during, and after the surgery allow a more optimal surgical path because when they are prepared, they will respond favorably to incidents that occur. Similarly, efficient emotional preparation of the patients will stimulate their willingness to collaborate in whatever is required and to their self-determination to recover, while enhancing the nurse-patient relationship, which lasts throughout the hospitalization.^8^ Although the entire surgical process is not without risk, mechanisms and strategies are available that contribute to minimize them to facilitate a successful postoperative adaptation process, so that the postoperative days of the patients are only the necessary and the quality of this phase is adequate with timely, efficient, and satisfactory nursing interventions; because the longer the recovery period, the complications that arise can be greater. Considering the input on this topic pertinent and relevant to improve nursing care for surgical patients, achieve their well-being during hospitalization and the restoration of their autonomy, the objective was to relate the degree of dependency with the hospitalization time of patients intervened surgically in a regional hospital in Peru to promote proper management, according to their care needs. 

## Methods

*Study design and sample.* The study was analytical, cross-sectional and retrospective, with 380 patients treated in the surgery service of the Regional hospital Docente in Cajamarca, Peru. The study included all the patients of both sexes and of different age groups, who were admitted to this service from 01 January 2021 until 24 March 2021. Patients without information in any of the variables assessed were excluded. 

*Techniques and data collection instruments.* The documentary analysis was used as data collection technique; the demographic and clinical information of the patients was obtained from the record of daily care of the hospital surgery service, through a file that collected information on the study variables, like sex, age in years, age group (children up to 11 years of age, adolescents from 12 to 17 years of age, youth from 18 to 29 years of age, adults from 30 to 59 years of age, and elderly adults 60 years of age and over); referral service (operating room, emergency, outpatient clinics, other); surgical intervention specialty (surgery, traumatology, neurosurgery, urology, oncology, other); surgical intervention performed (appendectomy, cholecystectomy, exploratory laparotomy, open reduction and internal fixation, craniotomy, osteosynthesis, hernioplasty, other); date admitted to the service; days of hospitalization and degree of dependency of patients previously classified in the clinical history, according to the criteria established by the Peruvian health system (grade I, demand for minimal or limited care, low-complexity postoperative period; grade II, demand for partial care, postoperative digestive and biliary tract and of medium complexity; grade III, dependent on the assistance of nursing professionals to cover their physiological and comfort needs, major surgery postoperative; and grade IV, patients in critical situation and highly dependent on the assistance of nursing professionals, high-complexity postoperative). 

*Data collection procedure.* Written authorization was obtained from the head of the hospital's surgery service for data collection of the variables investigated; thereafter, the surgery service was visited daily during January and March of 2021 to collect the data, during schedules and in an environment that did not interrupt care activities, until concluding with the process. 

*Information analysis.* The univariate description was conducted through absolute and relative frequencies and confidence intervals for proportions at 95% and for the correlation between the degree of dependency and hospitalization time the Log Rank (Mantel-Cox) - Chi-square and Kaplan-Meier survival analysis were applied, with statistical significance of *p*<0.05, procedures carried out in the SPSS statistical software v. 26. 

*Ethical aspects.* The ethical principles of beneficence and justice were efficiently applied during the development of the research. Authorization was obtained from the head of the surgery service of the Regional hospital Docente in Cajamarca for the collection and use of information contained in the registry of daily care, only for research purposes. No identifying information was obtained from the users. Given that this was a study without direct interaction with the participants, not entailing risks and due to the lack of ethics committees in the region studied, the dispensability of their participation is justified.

## Results

The patients seen in the surgery service of the regional hospital were mostly males (53.4%), with mean age of 35.3 ± 20.9 years with age range from 1 to 90 years, with 38.2% being adults. The highest proportion of patients were referred from the operating room (64.7%), especially from the surgery specialty (66.6%); the most-frequent surgical interventions were appendectomy with 49.7%, cholecystectomy with 10.7%, exploratory laparotomy with 9.4%, and open reduction and internal fixation with 8.1% (Table 1). 

**Table 1:** General characteristics of 380 patients seen in the surgery service of a regional hospital

Variables	***n* (%)**	95% CI
Sex		
Masculine	203 (53.4)	48.4 - 58.4
Feminine	177 (46.6)	41.6 - 51.6
Age		
Children	52 (13.7)	10.2 - 17.2
Adolescents	40 (10.5)	7.4 - 13.6
Youth	83 (21.8)	17.6 - 26.0
Adults	145 (38.2)	33.3 - 43.1
Elderly adults	60 (15.8)	12.1 - 19.5
Service of referral		
Operating room	246 (64.7)	59.9 - 69.5
Emergency	116 (30.5)	25.9 - 35.1
Outpatient clinics	11 (2.9)	1.2 - 4.6
Other	7 (1.8)	0.5 - 3.1
Surgical intervention specialty		
Surgery	253 (66.6)	61.9 - 71.3
Traumatology	71 (18.7)	14.8 - 22.6
Neurosurgery	39 (10.3)	7.2 - 13.4
Urology	9 (2.4)	0.9 - 3.9
Other	8 (2.1)	0.7 - 3.5

**Figure 1:**
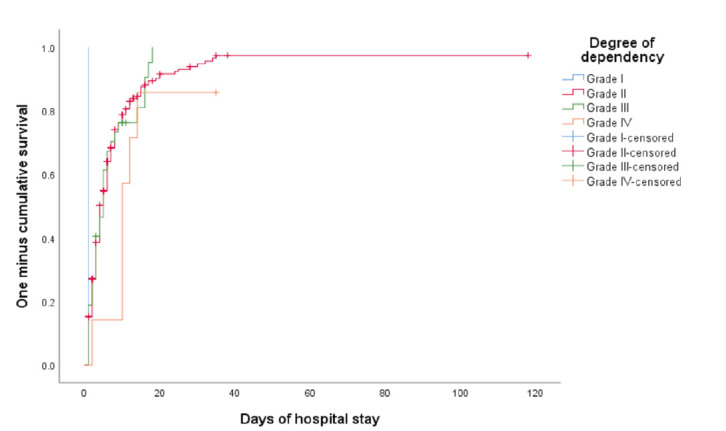
Kaplan-Meier survival analysis for patients intervened surgically

The mean hospitalization time of the patients was 10 days, with variations in function of their degree of dependency, with the number of days being greater in the highest degree of dependency (13 days); 88.1% of the patients had grade-II dependency, likewise, the degree of patient dependency had a significant impact on the days of post-surgery hospitalization (*p*=0.038) (Table 2), plotted in Figure 1. 

**Table 2:** Degree of dependency and hospitalization time of patients seen in the surgery service of a regional hospital

Degree of postoperative dependency	n	Surgical patients	Mean (days of hospitalization)	95% CI
Grade I	1	1	1.0	1.0-1.0
Grade II	335	269	9.9	6.9-12.9
Grade III	37	32	6.7	4.7-8.6
Grade IV	7	6	13.3	6.2-20.3
Global	380	308	10.1	7.3-12.8

* Log Rank (Mantel-Cox) - Chi-square: *p* = 0.038

## Discussion

The study results show similarities and differences shared with data reported in other sociodemographic settings, coinciding in that males were those most-often subjected to surgical interventions in Afghanistan (58.7%),^9^ the United Kingdom (53.6%),^10^ and Cuba (52.6%);^11^ in the United States, women (54.4%) surpassed men.^12^ These slight differences between sexes may be due to the random samples formed, according to the purpose of the studies, because there are nosological entities characteristic of each person, which could make a notable difference. Regarding age, the results are similar to those found in Afghanistan (mean age of 36.5 years)^9^ and the United Kingdom (mean age of 38.1 years),^10^ partly resembling the results in Cuba (from 18 to 35 years of age: 30.1%),^11^ but different from those in the United States (65 years of age and over: 65%).^12^ On this indicator, a lot will depend on the disease burden of each country, however, some diseases have a common demographic profile that can be the convergence point. 

With respect to the most-practiced surgical interventions, the results herein are related to that reported in Afghanistan where appendectomy had 72.8% of cases,^9^ in the United Kingdom with 69.1%,^10^ and in Cuba with 25% of cases.^11^ As the second most-performed surgery, cholecystectomy stood out in the United States (29.2%)^12^ and Cuba (14.8%).^11^ Unlike this study, the mean hospitalization time of patients in the United Kingdom was three days.^10^ Acute appendicitis is the most frequent clinical manifestation of acute abdomen in different contexts, which generally requires an appendectomy as treatment, does not pose major risks, and the recovery period is relatively short with effective assistance of essential nursing care, nonetheless, some complications can arise that prolong the hospital stay and compromise the patient’s recovery. 

Although the proportion of men seen in the surgery service is slightly above that of women, these can have a higher burden of comorbidities that make the difference, among them smoking, cardiac ischemia and arrhythmia, diabetes mellitus, liver disease, and cancer; likewise, the figures indicate that in men the diagnosis of pathologies occurs during advanced and complicated stages, as it happens in gallbladder diseases; however, women have a higher risk of mortality, above all when the surgeries are performed to repair damage caused by cancer. In this regard, differential treatment is necessary during the diagnosis, planning, and execution of surgical interventions.^13^^-^^15^ Similarly, contextualized knowledge by nursing professionals of the distribution of diseases with surgical outcomes according to sex, will allow for timely identification of risk factors, not only for better organization of the care they must provide, but to also lessen the impact on patients from a presurgical phase. 

Six of every ten participants corresponded to youth or adult age groups, ages in which some pathologies of acute surgical abdomen are prevalent, like acute appendicitis and cholecystitis, which are manifested as result of eating disorders or unhealthy habits accumulated over time, that is, deficient consumption of fruits and vegetables, excessive consumption of saturated fats and alcoholic beverages, insufficient physical activity, obesity, preexisting conditions, among others. Also, demographic changes over time show an increasing tendency of a greater number of cases of acute appendicitis with advancing age, which translates into a greater risk of complications and a more difficult reversal of the pathology. The outcome of these conditions generally leads to surgical removal of the affected organs and requires the assistance of grade II or III nursing care.^16^^-^^18^ Both pathologies are preventable if people's lifestyles are changed and to achieve such, constant health education is required whenever nursing professionals have contact with individuals. 

The surgery that was performed on half of the participants was the appendectomy, both open and laparoscopic. The latter has greater benefits because it is a less-invasive procedure, causes les pain in patients, occurrence of infection at the surgical site is reduced, patients require shorter hospitalization periods, the reincorporation to their daily life activities is undertaken without major implications, with an intact level of independence and with limited assistance from nursing care; therefore, in general terms, it is considered the safest technique, but both must be analyzed according to the characteristics of the patient and the availability of the human resources that provide care.^19^^-^^21^ Without risks and without complications, these aspects will determine an ephemeral hospitalization of patients, however, nursing professionals must be prepared for all possible scenarios upon knowing the surgical intervention trends and the care needs of patients, according to their degree of dependency, to optimize care and ensure the speedy recovery of those affected. 

In turn, the physiological response and post-surgery adaptation of each patient is different and the days of hospitalization may vary, as in the study, which had a mean of 10 days when considering all the surgical procedures performed, with greater demand for nursing care in grade-II patients, followed by grade III patients, although the latter with fewer days of hospitalization, which could be attributed to the proportional differences of both groups, to factors like age and existence of comorbidity conditions that determined the organic responses. Likewise, during surgical interventions, unexpected situations may arise that delay their resolution and encourage modifying the surgical procedure initially established, which, added to the physical, psychological, and demographic characteristics of the users, determine the post-surgery process. Aspects that have a considerable impact on rehabilitation time and patient survival,^6^^,^^22^^-^^24^ more so when the nursing resource is scarce or when the care needs exceed their working hours.^4^ Situations that must be addressed by those responsible of the health sector for better management of the nursing resource and of their clinical practice, which will contribute to increased patient satisfaction. 

Patient mobility, functionality, and autonomy are compromised after surgery; therefore, it is essential for body mobilization to begin 120 minutes after its completion and that necessary assistance be provided to improve respiratory function and oxygenation of the different organs, besides contributing to patients being less dependent and improving their quality of life,^25^^,^^26^ given that the degree of dependency influences significantly on days of hospitalization, so much so that the more dependent patients are, the longer time is required for their recovery and incorporation into their daily activities, given that their needs are complex and their situation is critical, and nursing care must be more assiduous and cautious, with establishment of priorities and more accurate diagnoses.^27^ Moreover, introducing pre-habilitation activities at physical and emotional levels acts as a fundamental pillar *a priori* for the overall success of the surgery.^28^ Results will not only be reflected at patient level, but also, the care provided will be of quality, health costs and the hospital offer will be more efficient and sustainable. 

One of the principal limitations of this study was the use of secondary sources that could have incomplete information on the study variables. 

In conclusion, the degree of patient dependency had a significant impact on the days of post-surgery hospitalization. The clinical-demographic profile presented by the patients serves as reference to establish and implement improvement plans in care before, during, and after a surgical intervention. Estimating the more-frequent surgeries, their characteristics, and number of hospitalization days will permit more effective hospital management. On the other hand, to strengthen the line of research in the context studied, research is required to evaluate the quality of life of patients during different periods after surgery. 

The correlation between the degree of patient dependency and days of hospitalization constitutes an important contribution to planning and redistributing human and material resources in institutions, based on patient categorization according to their care needs and time required for their resolution. It will also allow the provision of sufficient and qualified nursing professionals to cover the demand for care assistance with quality and efficiency. Implementing a continuous monitoring system in this sense could improve care and patient satisfaction, which can be evaluated in other research for their feedback.
